# A Recipe for Soft Fluidic Elastomer Robots

**DOI:** 10.1089/soro.2014.0022

**Published:** 2015-03-01

**Authors:** Andrew D. Marchese, Robert K. Katzschmann, Daniela Rus

**Affiliations:** Computer Science and Artificial Intelligence Laboratory, Massachusetts Institute of Technology, Cambridge, Massachusetts.

## Abstract

This work provides approaches to designing and fabricating soft fluidic elastomer robots. That is, three viable actuator morphologies composed entirely from soft silicone rubber are explored, and these morphologies are differentiated by their internal channel structure, namely, ribbed, cylindrical, and pleated. Additionally, three distinct casting-based fabrication processes are explored: lamination-based casting, retractable-pin-based casting, and lost-wax-based casting. Furthermore, two ways of fabricating a multiple DOF robot are explored: casting the complete robot as a whole and casting single degree of freedom (DOF) segments with subsequent concatenation. We experimentally validate each soft actuator morphology and fabrication process by creating multiple physical soft robot prototypes.

## 1. Introduction

**FIG. 1.**
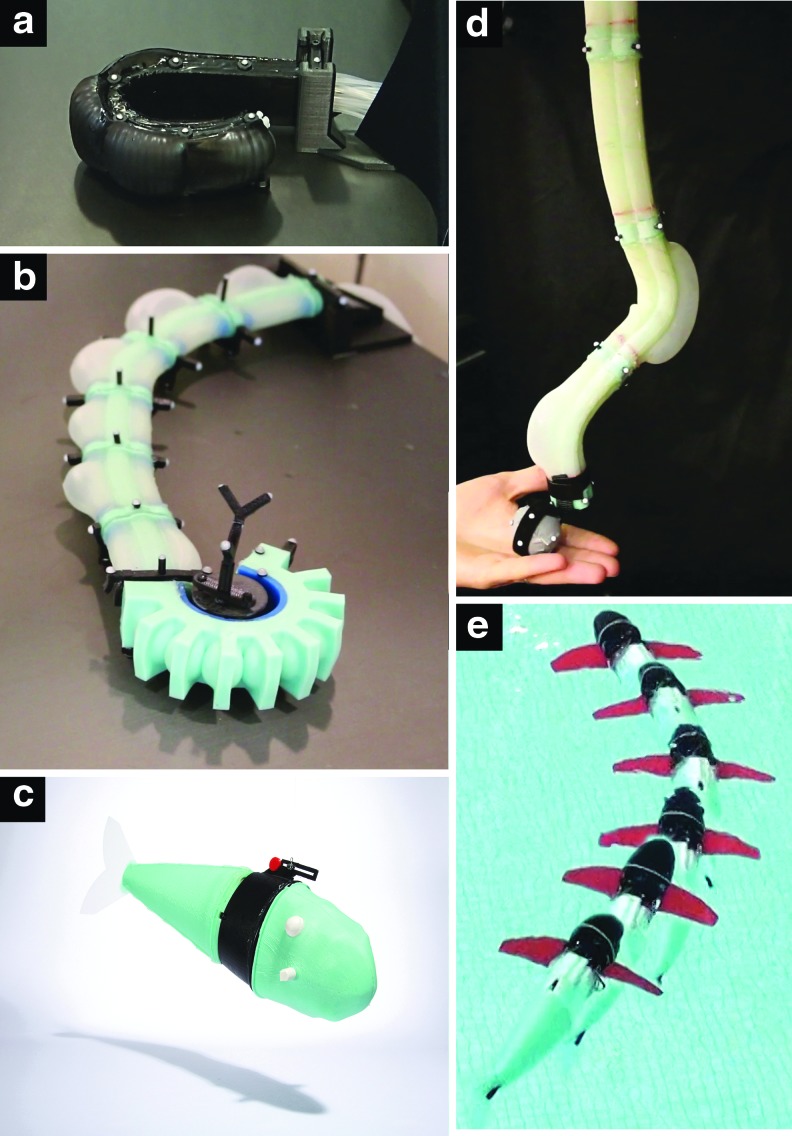
Extremely soft and highly compliant fluidic elastomer robots. **(a)** Ribbed planar manipulator.^8^ **(b)** Cylindrical manipulator with gripper.^11^ **(c)** Self-contained pneumatic fish.^46^ **(d)** Spatial cylindrical manipulator.^48^ **(e)** Self-contained hydraulic fish.^9^ Photo in panel **(c)** courtesy of Devon Jarvis for *Popular Mechanics*. Color images available online at www.liebertpub.com/soro

The goal of this work is to describe several fabrication methods for various kinds of soft robots. Each fabrication method produces one or several unit-modules that can be actuated based on the soft fluidic elastomer model. Each fabrication process can be used to create actuatable soft modules; these modules can be composed in series or in parallel to create a range of different soft robot morphologies. We experimentally validate these morphologies in the context of extremely soft and highly compliant locomotory robots and manipulators, as shown in Figure 1.

Soft robots exhibit continuum body motion, large-scale deformation, and relatively high compliance compared to traditional rigid-bodied robots.^1^ Such characteristics give this class of robots advantages like the ability to mitigate uncertainty with passive compliance,^2^ perform highly dexterous tasks,^3^ and exhibit resiliency.^4^ This work provides a recipe for designing and fabricating soft fluidic elastomer actuators and robotic systems.

Recent reviews articulate the challenges associated with creating robots from soft, nonlinear materials.^1,5–7^ Current engineering tools are well-suited for rigid-bodied robots, and when soft, nonlinear elastic materials are introduced, many of the underlying assumptions of these tools are not valid anymore. To create fluidic elastomer robots, we must overcome many technical challenges: (i) We need methods for composing soft-unit modules to create complex morphologies suitable for robot bodies capable of autonomous locomotion and manipulation. That is, we need to identify appropriate modules and ways of assembling these into multibody robots. (ii) Consistently reproducing certain properties of soft robots—for example, their elasticity or internal channel geometry—is difficult using conventional fabrication techniques. Accordingly, we must develop fabrication techniques that balance the competing goals of scalability and repeatability with the need for complicated features and shape profiles.

This work makes the following contributions:

1. Classification of three viable fluidic elastomer actuator (FEA) morphologies; that is, an FEA with a (i) ribbed channel structure and embedded transmission lines, (ii) cylindrical channel structure and hollow interior, and (iii) seamless pleated channel structure.
2. Three fabrication processes to reliably manufacture these FEAs. These are (i) a lamination-based casting process with heterogeneous embedded components, (ii) a retractable-pin-based casting process, and (iii) a lost-wax-based casting process.
3. A survey of recent robots built using these design and fabrication approaches.

This work significantly extends four previous conference publications: References^8–10^ and^11^.

This article is organized as follows. First, we review relevant soft actuation technology, design tools, and fabrication processes in section 2. Next, we present the design and characterization of three fluidic elastomer actuator morphologies in section 3. These actuator morphologies are differentiated by their internal channel structure, namely, ribbed, cylindrical, and pleated. Next, we provide three alternative fabrication approaches for reliably fabricating soft actuators and multisegment robots in section 4. These processes are lamination-based embedded casting, retractable-pin-based casting, and lost-wax-based casting. Then, we briefly discuss alternative approaches to powering these robots in section 5. And lastly, we demonstrate how the various actuator morphologies and fabrication processes have been used to realize a variety of soft autonomous systems: locomotory fishlike robots in section 6 and robotic manipulation systems in section 7.

## 2. Related Work

This article builds on several recent results in the design and fabrication of soft robots; see references^12–14^ for detailed reviews.

### 2.1. Actuation

**FIG. 2.**
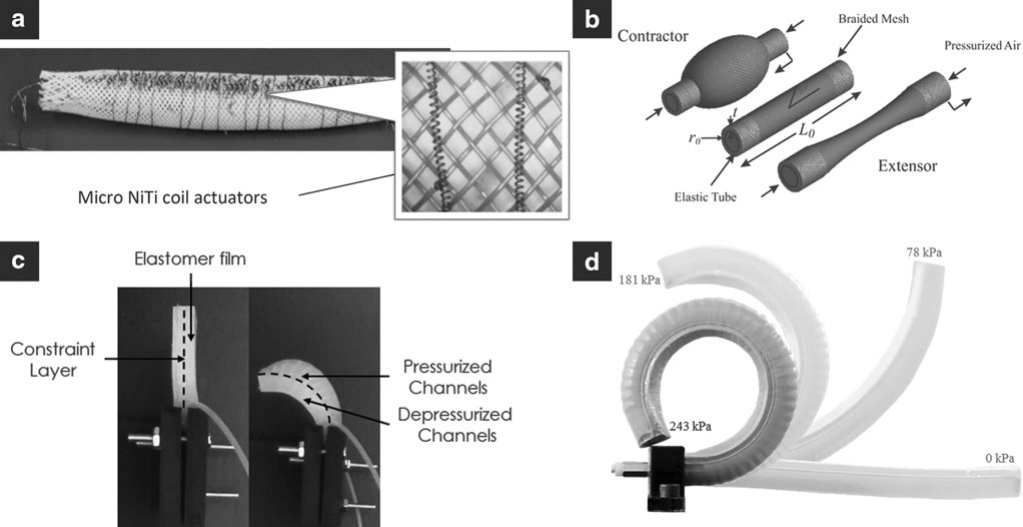
Common actuation approaches for soft robots. **(a)** Shape memory alloy (SMA) actuators.^18^ **(b)** Pneumatic artificial muscle (PAM) actuators.^2^ **(c)** Fluidic elastomer actuators (FEAs).^43^ **(d)** Fiber-reinforced FEAs.^58^

There are various approaches to actuating the body of a soft robot. One distinguishing feature of many soft robots is that actuators and/or power transmission systems are integrated within and distributed throughout the body. In the following, we review four common actuator types, and these are also depicted in Figure 2.

#### 2.1.1. Shape memory alloy actuators

The basic operating principle behind shape memory alloy (SMA) technology is that nickel titanium (NiTi) wire contracts under joule heating. This heating is typically produced by passing electrical currents through the wire. The contracting wire can be used as an agonist actuator, similar to the way one's bicep pulls the forearm toward the body. Kim *et al.* model, design, and fabricate these actuators and show their viability in soft robot applications.^15^ Additionally, the elastomer-based bioinspired octopus arms developed in Laschi *et al.* and Cianchetti *et al.* use SMA actuation to emulate a muscular hydrostat.^16,17^ Further, Seok *et al.* use SMA spring actuators to generate peristaltic locomotion in a wormlike robot^18^ (see Fig. 2a), Koh and Cho develop SMA coil-spring actuators to generate two-anchor crawling in an inchwormlike robot,^19^ and Umedachi *et al.* use SMA actuators to produce both crawling and inching in a 3D-printed soft robot.^20^

#### 2.1.2. Cable actuators

Originally, many hard hyper-redundant and hard continuum robots used an array of servomotors or linear actuators to pull cables that move rigid connecting plates located between body segments.^21–26^ Some softer robots have adopted a similar actuation scheme consisting of tendons pulling rigid fixtures embedded within an elastomer body. For example, the soft-bodied fish developed by Valdivia y Alvarado and Youcef-Toumi,^27^ the soft octopus-inspired arms developed in Calisti *et al.,*^28,29^ and the soft arm developed by Wang *et al.*^30^ use this actuation approach.

#### 2.1.3. Pneumatic artificial muscles

Another common actuation scheme for soft robots involves distributed pneumatic artificial muscle (PAM) actuators, also known as McKibben actuators, as shown in Figure 2b. A PAM is fundamentally composed of an inflatable elastic tube surrounded by a braided mesh. Depending on the weave pattern of the braided mesh, the actuator can be designed to contract or extend under input pressure. Typically, these actuators are operated with driving pressures between 50 to 100 psi. These actuators have been used and studied extensively in Chou and Hannaford,^31^ Tondu and Lopez,^32^ Caldwell *et al*.,^33^ Daerden and Lefeber,^34^ and Reynolds *et al.*^35^ Notable semisoft robots using PAMs include McMahan *et al.*,^2^ Pritts and Rahn,^36^ and Kang *et al.*^37^

#### 2.1.4. Fluidic elastomer actuators

A softer alternative is the fluidic elastomer actuator (FEA), which is used predominantly throughout this article (see Fig. 2c). The FEA is an actuator composed of low durometer rubber and driven by relatively low-pressure fluid in the range of 3 to 8 psi. Although many motion primitives are achievable with an FEA (*e.g.*, extending, contracting, twisting, and bending) in this work, we primarily focus on actuators designed for bending. Its basic structure consists of two soft elastomer layers separated by a flexible, but relatively inextensible, constraint. The inextensible constraint is typically created using cloth, paper, plastics, and even stiffer rubbers. Each of these elastomer layers contains embedded fluidic channels. By pressurizing the fluid entrapped in these channels, stress is induced within the elastic material producing localized strain. This strain in combination with the relative inextensibility of the constraint produces body segment bending. FEAs can be powered pneumatically or hydraulically.

As the review by Rus and Tolley discusses,^13^ perhaps the earliest application of pneumatically actuated elastomer bending segments to robotics was by Suzumori *et al.*^38^ Here, fiber-reinforced flexible microactuators (FMAs) were developed and shown to be viable in a manipulator and multifingered hand. Recently, these concepts have been extended and developed into the FEA and used to build a variety of soft mechanisms^39–42^ and soft robotic systems.^4,8–11,43–50^ Furthermore, Polygerinos *et al.* and Mosadegh *et al.* have investigated more elaborate channel designs in order to reduce elastomer strain on the outer layer of the actuator, allowing for higher bending curvatures.^51,52^ Additionally, Cianchetti *et al.* develop a fluid actuated bending arm^53^ with a jamming spine.^54,55^

There are also less flexible, fiber-reinforced FEAs (see Fig. 2d) that occupy the soft actuator space between purely elastomer FEAs and PAMs. While these actuators have to operate with comparably higher driving pressures between 25 and 35 psi, they can accordingly apply higher forces, which is advantageous for certain applications. There are several notable examples of fiber-reinforced FEAs in the literature.^3,38,56–60^

### 2.2. Design tools

Design tools for soft robots are limited with respect to the availability of design tools for more traditional rigid-body robots. Suzumori *et al.* use finite element modeling (FEM) to analyze the bending of fiber-reinforced pneumatic tubelike actuators.^56^ Specifically, hyper-elastic material models are used to capture the nonlinear material properties of rubber, line elements are used to represent radial inextensibility constraints due to fiber reinforcement, and the simulation is performed using the software MARC. Outside of this example, the community has generally found that iterative nonlinear finite element solvers are limited to small deformations and of limited use when modeling very soft nonlinear materials.^6^ VoxCAD and the Voxelyze physics engine, as used in Cheney *et al.*^61^ and Lehman and Stanley,^62^ and reviewed by Lipson,^6^ are simulation tools for very soft nonlinear materials. These tools use the concept of nonlinear relaxation to effectively perform physically correct particle-based material simulation. They have the advantage of allowing the user to individually set the local material properties of each particle. The disadvantage is that many physical parameters of active and passive material types must be experimentally derived.

### 2.3. Fabrication

**FIG. 3.**
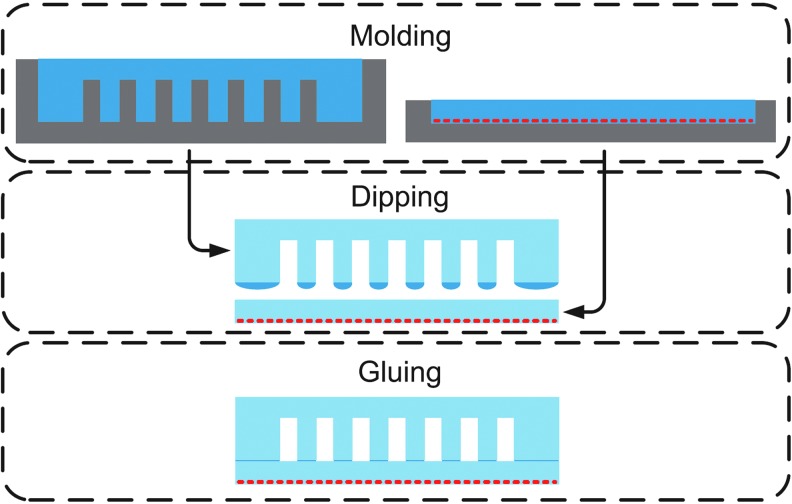
Soft lithography fabrication process for soft fluidic elastomer robots. Reproduced with permission from Onal and Rus.^74^ Color images available online at www.liebertpub.com/soro

Cho *et al.* review several manufacturing processes for soft biomimetic robots.^63^ The vast majority of soft elastomer robots rely on the processes of soft lithography^64^ and/or shape deposition manufacturing.^65^ Specifically, for soft fluidic elastomer robots this fabrication process generally consists of three steps, as shown in Figure 3: (1) Two elastomer layers are molded through a casting process using pourable silicone rubber. The mold used for the outer layer contains a model of the desired channel structure. When cast, the outer layer contains a negative of this channel structure. The mold used for the constraint layer may contain fiber, paper, or a plastic film to produce the inextensibility property required for actuation. When the elastomer is poured, this material is effectively embedded within the constraint layer. (2) The two layers are cured, removed from their molds, and their joining faces are dipped in a thin layer of uncured elastomer. (3) Lastly, the two layers are joined and cured together. The primary limitation of this soft lithography fabrication process is that it is fundamentally 2.5D, meaning that the robots are largely constrained to a planar morphology. This process limits a soft robot's ability to achieve amorphous, 3D forms. Additionally, Umedachi *et al.* provide the first SMA actuated soft robot fabricated using 3D printing.^20^ However, although 3D printing allows printing flexible materials in amorphous forms, these materials are relatively brittle with respect to casted rubbers and are therefore not well-suited for FEAs, which rely on pressurization of the rubber.

### 2.4. Soft locomotory robots

In the past years, soft roboticists have made many notable low durometer rubber robots intended for land and water locomotion. For example, rolling belts have been produced by Correll *et al.*^66^ and Marchese *et al.*^44^ Trimmer *et al.* and Umedachi *et al.* emulated the peristaltic locomotion of caterpillars.^20,67^ Shepherd *et al.* developed a multigait walking robot,^39^ and Shepherd *et al.* developed a jumping robot powered by combustion.^68^ However, a limitation of the aforementioned locomotory robots is that they require an electrical and/or pneumatic tether. Soft actuation systems, especially fluidic actuation systems, typically require significant supporting hardware and often limit soft locomotory robots from being self-contained. That said, there are a few examples of untethered soft robots: Onal *et al.* created a rolling robot,^43^ Onal and Rus emulated the serpentine locomotion of snakes^45^ and Tolley *et al.* developed a quadrupedal walking robot;^4^ these are all soft-bodied fluidic elastomer systems. Seok *et al.* realize peristaltic locomotion with a self-contained SMA-based inchworm.^18^ However, a limitation of all these untethered soft platforms is that performance is severely limited with respect to their rigid-bodied counterparts, and this limitation is due to the constraints imposed by bringing onboard all supporting hardware. More specifically, they all exhibit locomotory speeds of between 0.008 and 0.07 body lengths per second. Recently, Marchese *et al.* developed an autonomous soft robotic fish that can perform escape maneuvers with speeds up to 0.4 body lengths per second.^46^ Katzschmann *et al.* presented a soft fish that can swim in 3D for prolonged periods of time and powers its FEA tail hydraulically.^9^

### 2.5. Soft continuum manipulators^*^

Recently, continuum manipulators composed from soft elastic material have been developed. These soft rubber manipulators can be categorized under two primary morphologies. The first morphology type are tendon-driven manipulators consisting of variable length tendons, typically cables or shape memory alloy wire, embedded within and anchored to portions of a soft silicone rubber arm. For example, previous work on soft bioinspired octopuslike arms developed by Calisti *et al.*^28^ used tendons and demonstrated capabilities like grasping and locomotion.^16,29^ Also, Wang *et al.* developed a cable-driven soft rubber arm consisting of one large actuated segment that bends bidirectionally.^30^ Lastly, McEvoy and Correll used a programmable stiffness spine in conjunction with tendons to achieve shape change in a soft rubber arm.^69,70^ The second morphology uses fluidic elastomer actuators (see section 2.1.4) distributed among the manipulator's soft body segments. The primary advantages of using fluidic actuation for soft continuum manipulators is that this energy transmission system: (i) can be lightweight, making for easy integration into distal locations of the body; (ii) conforms to the time varying shape of the manipulator; and (iii) does not require rigid components to implement. There are several examples of soft fluidic grippers described in recent literature. Deimel and Brock developed a pneumatically actuated three-fingered hand made of fiber-reinforced silicone that is mounted to a hard industrial robot and capable of robust grasping.^59^ More recently, they have used similar fiber reinforced actuation technology to develop an anthropomorphic soft pneumatic hand capable of dexterous grasps.^3^ Additionally, we have previously shown planar manipulation is possible with an entirely soft robot. That is, a six-segment planar fluidic elastomer robot can be precisely positioned using a closed-loop kinematic controller.^8,10,11^ Ilievski *et al.* created a pneumatic starfishlike gripper composed of FEAs and demonstrated it grasping an egg.^40^ Stokes *et al.* used an FEA-based elastomer quadrupedal robot to grasp objects in a hard–soft hybrid robotic platform.^71^ A puncture resistant soft pneumatic gripper is developed in Shepherd *et al.*^72^ An alternative to positive pressure actuated soft grippers is the robotic gripper based on the jamming of granular material developed in Brown *et al.*^54^ Another relevant piece of work is the manually operated 3D elastomer tentacles developed by Martinez *et al.*, containing nine pneumatic crescent-shaped channels embedded within three body segments.^42^

## 3. Actuators

In this section we detail the design and fabrication of three different soft fluidic elastomer body segments. Each type of body segment can serve as a unit-module for composing different soft robot body morphologies. The primary design constraint is that the actuated body segments should be composed almost entirely from soft materials. The primary functional specification is that these actuated segments should integrate into an autonomous robotic system. That is, they should be capable of performing tasks such as trajectory-following in free space, moving dexterously through confined spaces, and/or grasping and placing objects, all without human intervention.

### 3.1. Operating principles

**FIG. 4.**
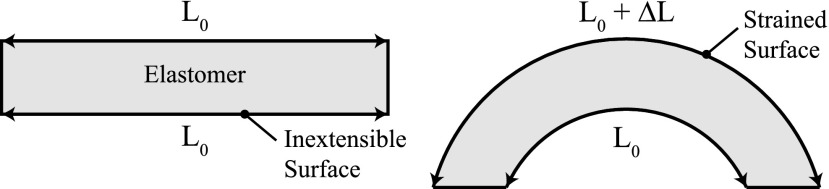
Operating principle of a bending elastomer segment. One surface of the elastomer is strained, while the opposite side remains unextended. The difference in length produces bending.

Despite the variability in fluidic elastomer actuator morphologies, their fundamental operating principles are universal. This section provides an overview of these operating principles. Generally, each segment of a fluidic elastomer robot bends, and this bending is due to material strain. Figure 4 illustrates how unidirectional bending arises from material strain. Consider a block of elastomer where the edges of the top and bottom surfaces have equal lengths, *L*_0_. If the top surface is strained such that its new edge length is *L*_0_+Δ*L*, but the bottom of this block remains unextended, then the elastomer will bend. Bending is the basic motion primitive of the fluidic elastomer robot.

**FIG. 5.**
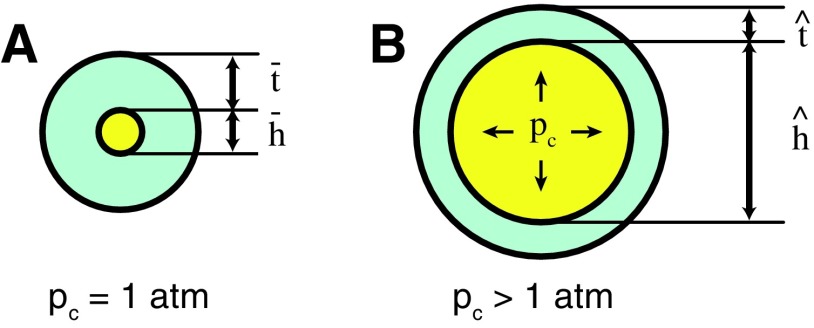
Operative principle of producing material strain through fluidic power. **(A)** Fluid, shown in yellow, is entrapped in an elastomer channel. **(B)** When the fluid is pressurized, stress and therefore strain are generated in the material.

In order to generate strain within the elastomer, this class of actuator uses pressurized fluids. Essentially, expandable, fluid-filled chambers are embedded within the elastomer. When these chambers are pressurized, the entrapped fluid generates stress in the material, causing the material to strain. This concept is illustrated in Figure 5A and B. Here, the entrapped fluid is shown in yellow and its pressure is *p_c_*. In order to express the relationship between fluid pressure and elastomer deformation, we can use a one-dimensional simplification of an iterative model, as presented in Marchese and Rus.^48^ Let $\bar{h}$ and $\,\bar { t}$ be the initial undeformed diameter and wall thickness of a cylindrical elastomer channel, and let $\hat { \bf h}$ and $\hat { \bf t}$ represent the deformed diameter and wall thickness. Algorithm 1 expresses how the channel's diameter grows as a function of pressure. Stresses are successively updated based on deformed channel dimensions. Here, Δ**p**_***c***_ is a vector of all consecutive incremental pressure increases until the maximum channel pressure $p_c^{{ \max}}$ is reached. The stress and strain in the elastomer are represented by σ and ε, respectively. The procedure strainLookUp () provides a nonlinear mapping from stress to strain.

**Algorithm 1:** Iterative Channel Deformation

### 3.2. Actuator morphologies

This section provides an in-depth look at three separate soft elastomer body segments actuated using pressurized fluids. We use a defining structural feature to refer to each of the presented segment morphologies, those are (i) ribbed, (ii) cylindrical, and (iii) pleated. In section 7, these segments are combined serially to form multibody manipulators, and in section 6 they are used to form single and multibody locomotory robots. Although similar in material composition and function, differences in internal and external structure and form lead to several distinct differences between the three presented morphologies. First, we present each morphology, examining the structural differences. Then, we provide a comparative characterization of the segments, highlighting salient performance characteristics.

#### 3.2.1. Ribbed segment

**FIG. 6.**
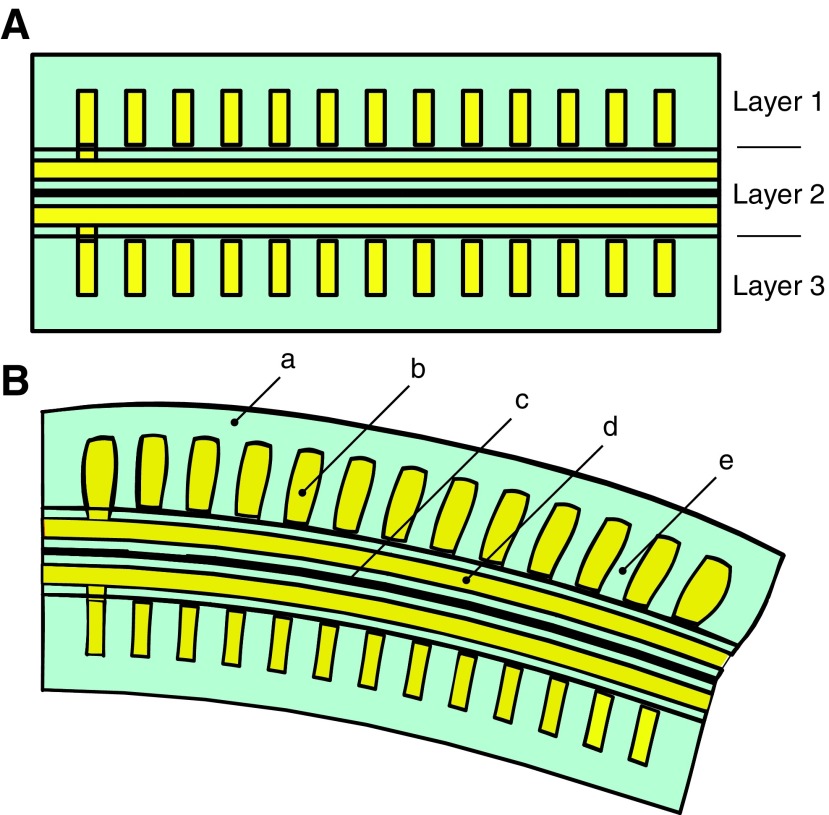
A conceptual representation of the ribbed segment morphology. The segment is composed of three layers produced from soft elastomer (a), embedded fluidic channels (b), inextensible, but flexible constraint (c), embedded fluid transmission lines (d), and ribbed structures (e). **(A)** The segment in an unactuated, or neutral state. **(B)** The segment in an actuated state where fluid within the agonist channel group is pressurized, producing bending about the inextensible axis. Color images available online at www.liebertpub.com/soro

The ribbed fluidic elastomer actuator with its multiple rectangular channels was first implemented and characterized in Correll *et al.*,^66^ and followed by Onal *et al.*^43^ and Onal and Rus.^45^ Joining two fluidic elastomer actuators in an agonist–antagonist pairing provides bidirectional bending. This actuator type provided the fundamental segment-level structure of the manipulator developed in Marchese *et al.*^8^ We refer to this three-layer composite here as a ribbed segment. That is, two actuator layers are combined in a pair but separated by an inextensible constraint layer. An implementation of this segment morphology is shown in both a neutral (Fig. 6A) and bent (Fig. 6B) state. Bending is produced through the pressurization of agonist fluidic channels (Fig. 6b) that are embedded within the actuated layers (Fig. 6, layers 1 and 3). The structure of the actuated layers is cast from soft elastomer (Fig. 6a). When pressurized, the agonist fluidic channels expand and strain the elastomer. This deformation is transferred into bending by means of an inextensible but flexible constraint (Fig. 6c) embedded within the center layer (Fig. 6, layer 2). Ribs located between channels (Fig. 6e) mitigate strain normal to the inextensible neutral axis. At the segment level, Marchese *et al.* extended the ribbed segment design to make it suitable for inclusion in a multisegment manipulator.^8^ Specifically, fluidic supply channels (Fig. 6d) were introduced on either side of the inextensible constraint and embedded within the center layer. Each segment accommodates multiple, parallel supply channels, two for each body segment within the manipulator. For a detailed model of how a ribbed segment deforms under fluidic pressure input, please refer to Marchese *et al.*^46^ It is important to note that this simplifying static model assumes that ribbed channels deform purely by extending their side and top walls, and that these wall stresses are based on initial channel geometry. In reality, as is shown here in Algorithm 1, wall stresses change as a function of the deformed geometry. If needed, Algorithm 1 can be used to augment the ribbed model with variable geometry used for the soft robotic fish in Marchese *et al.*^46^

**Pros:** The primary benefits of this morphology in relation to alternatives presented in this section are: (1) Ribs between channels mitigate strain normal to the neutral axis. (2) For a fixed fluid energy input, this segment exhibits greater bending than the cylindrical segment.

**Cons**: The primary disadvantages of this morphology in relation to alternatives presented in this section are: (1) The three-layer structure is prone to delamination and rupture under high strain. (2) Manufacturing this rectangular, layered structure is challenging because all transmission lines must be embedded within the thin constraint layer.

#### 3.2.2. Cylindrical segment

**FIG. 7.**
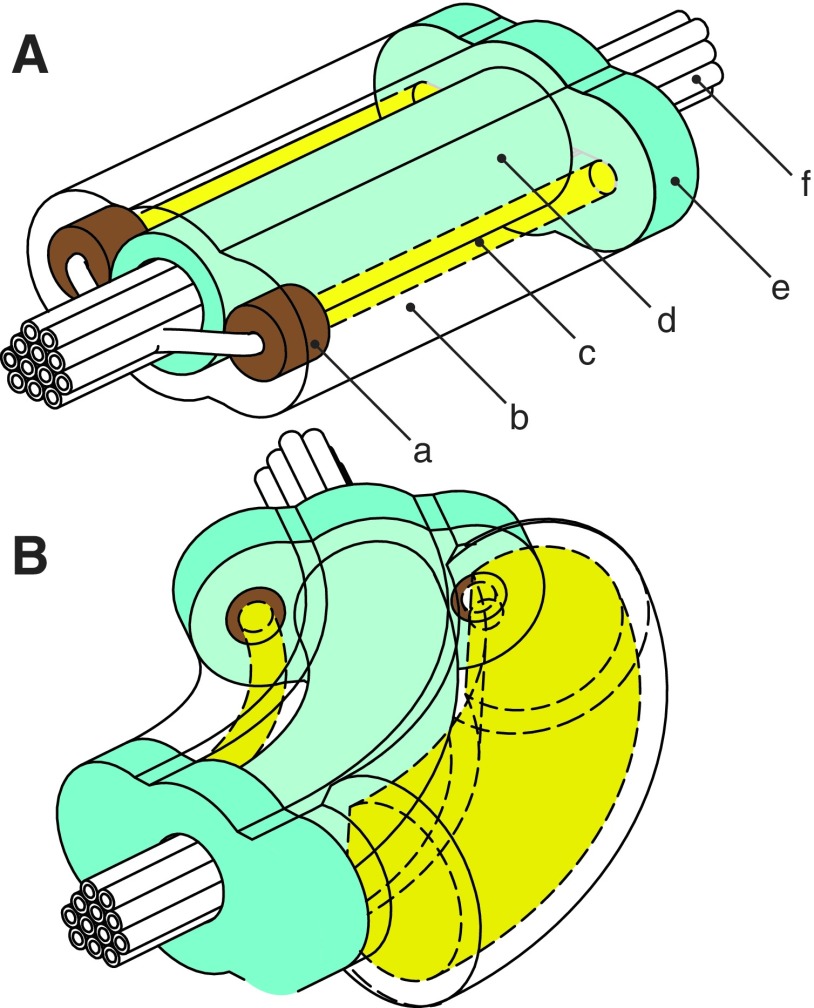
A conceptual representation of the cylindrical segment morphology. The segment consists of a soft silicone rubber outer layer (b, transparent), a slightly stiffer silicone inner layer (d, cyan), crush-resistant silicone inlets (a, brown), expanding embedded fluidic channels (c, yellow), and an internal tubing bundle (f, white). The segment terminates in soft endplates (e). **(A)** A depiction of the segment in an unactuated state. **(B)** A depiction of the body segment in an actuated state where the expansion of the pressurized fluidic channel is schematically represented.

The cylindrical fluidic elastomer segment is an alternative to the ribbed design. This design was first presented by Marchese *et al.*^10^ Design inspiration was drawn from the soft rubber tentacles developed by Martinez *et al.*,^42^ which use embedded crescent-shaped channels in a similar two-layer rubber construction. Although the cylindrical segment morphology is notably different from the ribbed segment, the fundamental operating principles are the same. In the cylindrical morphology (Fig. 7A and B), we transition from a rectangular, planar-layered composite to a cylindrical, concentric-layered composite. Specifically, the segment consists of three concentric layers: (i) an outer soft layer (Fig. 7b, *transparent*), (ii) a slightly stiffer inner layer (Fig. 7d, *green*), and (iii) a hollow core that accommodates a bundle of fluid transmission lines (Fig. 7f, *white*). Two fluid-filled and cylindrically shaped channels are embedded laterally within the outermost layer (Fig. 7c). These channels interface with the transmission lines by means of a stiffer rubber inlet piece (Fig. 7a, *brown*). When pressurized, the entrapped fluid deforms the embedded channel both circumferentially and longitudinally (Fig. 7B). Specific to this morphology, the inner tubelike layer composed of slightly stiffer rubber serves as an inextensible constraint, transforming channel deformation into segment bending.

**Pros:** The primary benefits of this morphology in relation to alternatives presented in this section are: (1) Entirely composed of rubber, the resiliency and the durability of the actuator are increased. (2) The two cylindrical channels make this segment the simplest to fabricate. (3) Embedded fluidic channels are not at the interface between fabricated layers, making this morphology robust against delamination under high pressures.

**Cons:** The primary disadvantages of this morphology in relation to alternatives presented in this section are: (1) The simple channel design exhibits high circumferential strain. Compared to the ribbed and pleated morphologies, more fluid energy is required to produce bending. (2) When the segment bends, an increased volume of rubber on the antagonist side of the actuator has to be compressed. This inhibits a high maximum curvature.

#### 3.2.3. Pleated segment

**FIG. 8.**
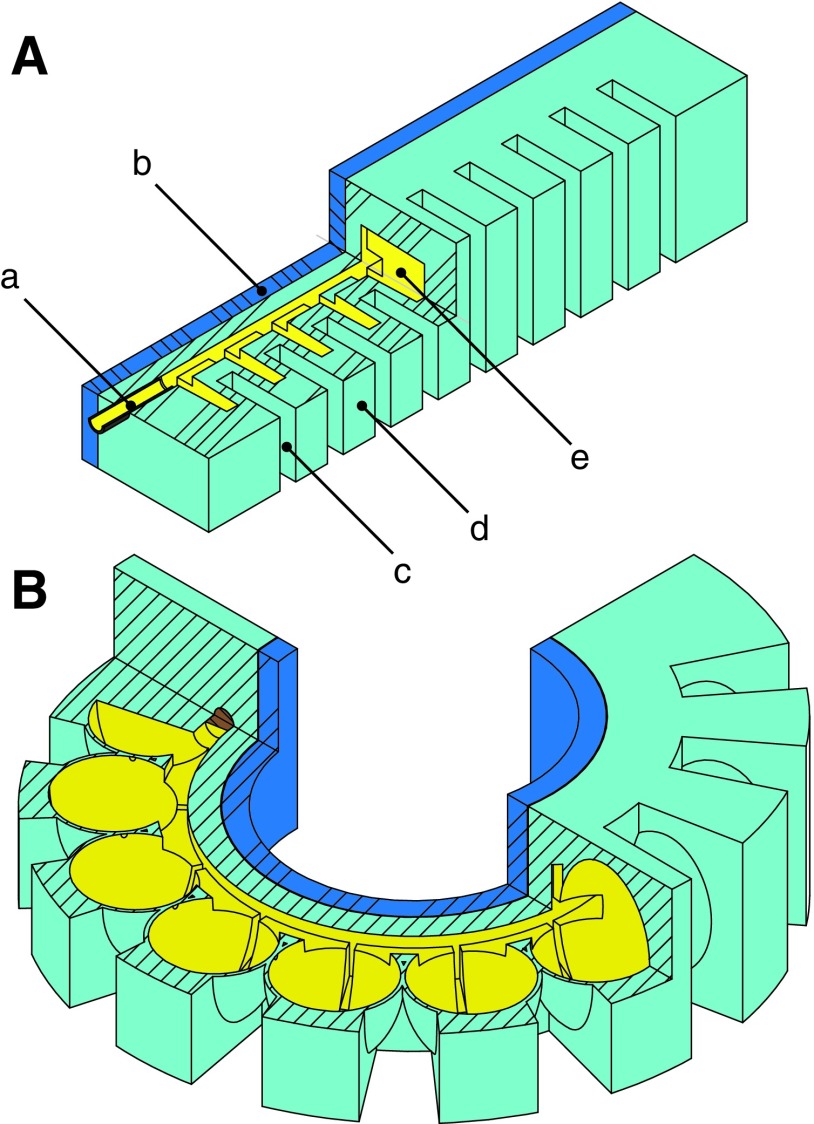
A conceptual representation of the pleated segment morphology. The design consists of a channel inlet (a), an almost inextensible constraint layer (b), uniform pleats (d) separated by even gaps (c), and internal channels within each pleat (e). **(A)** depicts the segment in an unactuated state and **(B)** shows the segment in an actuated and therefore bent state. The expansion of the pressurized channels is schematically represented.

The pleated channel design is detailed in Figure 8 and consists of evenly spaced, discrete elastomer sections (Fig. 8d), which are separated by gaps (Fig. 8c). Embedded within each elastomer section is a hollow channel (Fig. 8e). Cut views of the unactuated and actuated states are shown in Figure 8A and B, respectively. This design approach draws inspiration for its pleats from the soft pneumatic gloves developed by Polygerinos *et al.*,^51^ and its homogeneous body design is inspired from the tail design of a soft robotic fish developed by Katzschmann *et al.*^9^ The hollow channels within each pleat are connected via a center channel and are accessible through a front inlet (Fig. 8a). When fluid within these channels is pressurized (Fig. 8, *yellow*), an individual pleat undergoes a balloonlike expansion of the thin exterior skin, both normal and parallel to the neutral axis. Similar to the cylindrical actuator design, a stiffer silicone layer (Fig. 8, *blue*) serves as an almost inextensible constraint layer. The sum of the balloonlike expanding motions leads to bending of the less extensible center constraint layer.

**Pros:** The primary benefits of this morphology in relation to alternatives presented in this section are: (1) A unidirectional pleated actuator is capable of bending to higher curvatures than the ribbed or cylindrical morphology. (2) A bidirectional pleated segment is capable of exerting higher maximum forces because of its ability to accommodate the largest energy input. (3) Using a lost-wax casting approach, the *cyan* portion of this segment can be cured in a single step, avoiding seams that are prone to delamination.

**Cons**: The primary disadvantages of this morphology in relation to alternatives presented in this section are: (1) The morphology is more complex to manufacture because it requires a lost-wax casting procedure detailed in section 4.3. (2) The implementation of this morphology requires the most fluid energy to actuate it to appreciable tip forces. This might very well be due to the fact that, when compared to the other implementations, this implementation is larger in size and uses a higher shore hardness elastomer.

#### 3.2.4. Comparative characterization

To characterize the actuated segments, we first perform bending tests to experimentally determine the relationship between the segment's neutral axis bend angle *θ*, internal channel pressure *p_c_*, and supplied volume $p_c \ge p_c^{max}$ for each morphology.

**FIG. 9.**
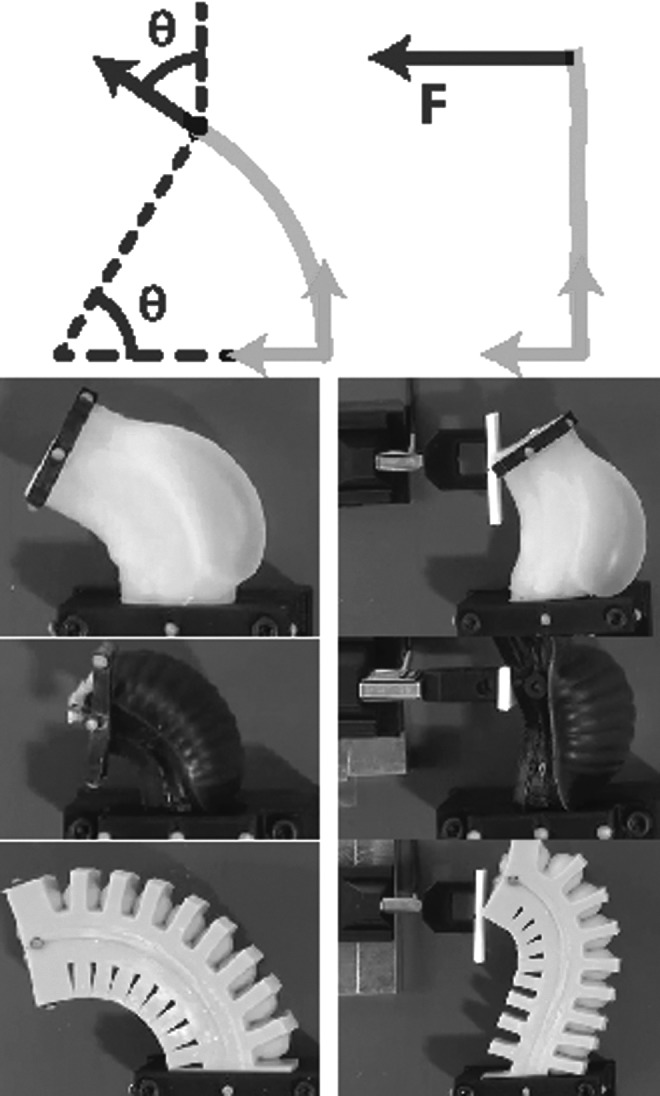
Experimental setup of the comparative characterization. Left column shows bend angle measurements. Right column shows blocking force measurements via a load cell.

In these experiments, the base of each segment is grounded securely in a fixture, and the segment's tip is supported vertically with a ball transfer. The setup is shown in the left column of Figure 9. The segment's agonist channel is incrementally filled under closed-loop volume control via the displacement of a fluidic drive cylinder; please refer to section 5. After each incremental fill, we allow pressure within the cylinder and within the actuated channel to equalize before measurements of the channel's pressure and the segment's curvature are taken. Curvature is assumed to be constant along the length of the segment and is uniquely defined by measuring the cartesian locations of the base and the tip of the segment; refer to Marchese *et al.*^8^ From this curvature we compute the segment's bend angle.

Since this is a quasistatic process, fluid pressure and supply volume measurements can be used to determine the elastic potential fluid energy input into the actuation system. The actuation system consists of the elastomeric segment and the internal compressible transmission fluid. The elastic potential fluid energy serves as a comparative metric between the different actuator segment designs. The potential energy is calculated by

\begin{align*}V_{Elastic} = \int_0^{{\mathbb V}_c} \ p_c \ ( {\mathbb V} ) \ { \rm d} {\mathbb V}. \tag{1}\end{align*}

Each segment's geometry and cavity volume is different, because each actuator segment was built with a different type of robot prototype in mind. The geometries and the resulting cavity volumes are listed in Table 1. The different cavity volumes and the different characteristic deformations of each morphology under pressurization require significantly different volumetric displacements.

**Table 1.** Geometric Parameters of an Actuator Segment

	*Actuator type*		
	*Ribbed*	*Cyl.*	*Pleated*
Actuator length [mm]	37.8	61.2	107.5
Actuator width [mm]	32.0	33.5	44.4
Actuator thick. [mm]	18.5	19.6	25.4
No. of channels per side	13	1	10
Single channel length [mm]	25.4	40.0	12.9
Single channel width [mm]	3.1	2.8	12.3
Single channel thick. [mm]	1.0	2.8	2.8
Cavity volume per side [ml]	1.04	0.31	5.12

Additionally, a blocking force test is performed in order to understand the variability in tip force output between segment morphologies. Again, a similar experimental procedure is used as for the bending characterization; however, during blocking force experiments a plate attached via a force transducer to ground is mounted in contact with the segment's tip, orthogonal to the bending plane. This effectively measures the force required to block the actuator from bending. The setup is shown in the right column of Figure 9.

**FIG. 10.**
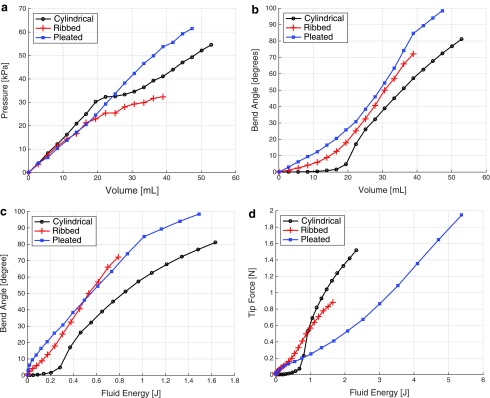
Experimental characterizations of three actuated segment morphologies performed by filling each actuator by means of controlled volumetric displacements and measuring internal pressure, neutral axis bend angle under a constant-curvature assumption, and blocking force.

Figure 10 details the results of these characterization experiments from which we can make several observations. First, the relationship $\frac { \partial p_c }  { \partial { \mathbb V } _c }$ is similar among the different morphologies for inputs up to approximately 20 mL. In the regime where ${\mathbb V}_c$ is above 25 mL, the pleated morphology has the highest $\frac { \partial p_c }  { \partial { \mathbb V } _c }$, followed by the cylindrical, and then the ribbed (Fig. 10a). Second, the cylindrical morphology has a salient bend angle nonlinearity (Fig. 10b). More specifically, small volumetric fluid changes of less than 15 mL provide little control authority over curvature; however, above 25 mL displacements, the control authority is strong and the curvature–volume relationship is approximately linear. This can be explained by the initial, relatively large radial expansion of the segment. Third, for a given fluid energy input, the bending angle of the cylindrical actuator is the least while the blocking force is the highest. In this morphology, a considerable amount of fluid energy radially expands the actuated channel. This energy does not contribute to axial expansion and therefore does not contribute to increasing the bend angle. However, the radial expansion causes a considerable increase in area moment of inertia, which stiffens the actuator and causes it to have a higher blocking force than the other designs. Fourth, the cylindrical morphology requires the most amount of fluid energy to produce a given bend angle, and the ribbed and pleated segments require approximately the same amount of fluid energy to generate equivalent bending (Fig. 10c). This observation holds over the range of inputs generated during these experiments. Last, the pleated segment requires more fluid energy than both the ribbed and cylindrical morphologies to produce a given tip force for inputs greater than 1 J. However, the pleated segment can accommodate significantly higher input energies and therefore can reach the highest maximum tip force. Each actuator was inflated to either its maximum before the elastomer plastically deformed or to the highest feasible bend angle. The pleated prototype is larger in scale than the cylindrical and ribbed; therefore, it can be driven to higher energy inputs.

## 4. Fabrication

Three distinct fabrication techniques for soft actuators are presented in this section. Table 2 contains the superscript references to machine tools and materials used.

**Table 2.** Commercially Available Tools and Equipment

*No.*	*Product name*	*Company*
1	Fortus 400mc	Stratasys
2	VLS3.50	Universal Laser Systems
3	Ecoflex 0030	Smooth-On
4	AL Cube	Abbess Instr. & Systems
5	Mold Star 15	Smooth-On
6	Silicone Sealant 732	Dow Corning
7	PN 51845K52	McMaster
8	PN 5742T51	McMaster
9	PN 51845K53	McMaster
10	Mold Star 30	Smooth-On
11	Beeswax	Jacquard
12	PN 2153T31	McMaster
13	PN 9808K21	McMaster

### 4.1. Lamination casting with heterogeneous embeddings

Lamination-based casting with heterogeneous embeddings is a fabrication technique that extends current soft lithography casting processes. As detailed in section 2.3 and in Figure 3, the outer layers of a soft robot are often cast separately using soft lithography techniques to inlay channel structures. Then, these layers are laminated together with a constraint layer to form the actuator. To power actuation, supply lines are pierced through the actuator's side wall and run external to the mechanism. This approach can be prohibitive in that it creates an unreliable pneumatic interface between supply lines and actuated channels, and also these external supply lines can inhibit the robot's movement or otherwise obstruct it from completing its intended function. By embedding heterogeneous components within the elastomer layers as they are cast, we address both of these challenges. In this section, we show how the idea of soft lithography can be combined with embedding heterogeneous components and that it is well-suited for realizing the ribbed body segment morphology. Specifically, we illustrate this fabrication process in the context of creating both a soft ribbed manipulator and soft ribbed fish robot.

**FIG. 11.**
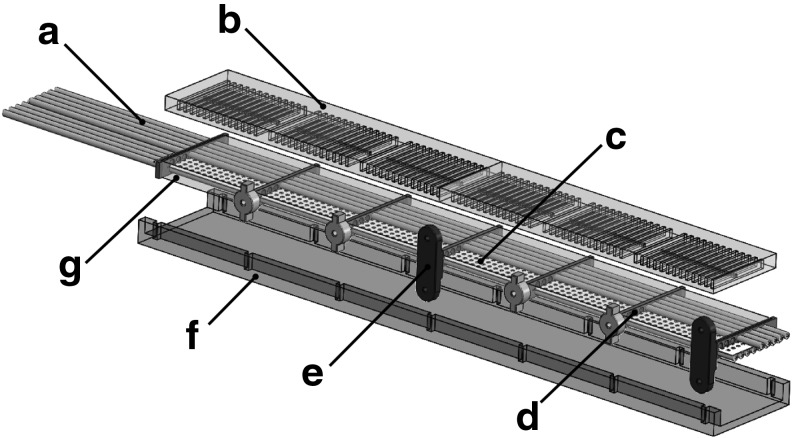
Fabrication process for a ribbed manipulator: silicone tubing (a), elastomer pieces containing channels (b), constraint film (c), constraint supports (d), feet (e), constraint layer mold (f), and composite constraint layer (g).

A ribbed manipulator, like that detailed in section 7.1, can be fabricated using lamination-based casting with heterogeneous embeddings. The specific approach for fabricating a six-segment manipulator is illustrated in Figure 11. Here, seven constraint supports (Fig. 11d) are 3D printed^1^ and placed into a constraint layer mold (Fig. 11f), which is also 3D printed. The constraint film (Fig. 11c) is cut from a thin acetal sheet^8^ using a laser^2^ and inserted through the aforementioned supports. Above and below the constraint film, eight pieces of silicone tubing (Fig. 11a) are threaded through the supports. Silicone rubber^3^ is then mixed and poured into the constraint layer mold, immersing tubing, film, and supports in a layer of elastomer to create the composite constraint layer (Fig. 11g). The uncured rubber inside the mold is then immediately degassed using a vacuum chamber.^4^ Once cured, small holes are created in the constraint layer to pierce the embedded tubing at specific locations, allowing each line to independently address a group of fluidic channels. Elastomer pieces containing channels (Fig. 11b) are casted and cured separately using a similar molding technique. Those cured elastomer pieces (Fig. 11b) are then carefully attached to both faces of the constraint layer using a thin layer of silicone rubber. Lastly, the printed feet (Fig. 11e) are attached to the constraint supports (Fig. 11d) to create an attachment point for ball transfers. These mechanisms help constrain the arm's motion to a plane.

**FIG. 12.**
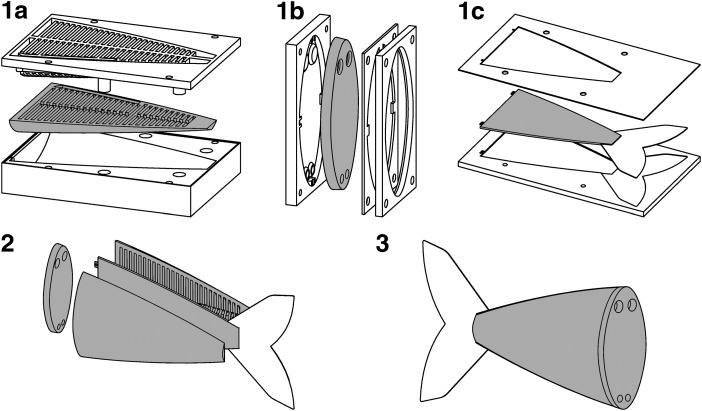
Illustration of the soft fish body fabrication process. First, two halves of the body **(1a)**, a connector piece **(1b)**, and a constraining layer **(1c)** are all cast from silicone using two-part molds. Next, these four pieces are sequentially bonded together using a thin layer of silicone **(2)**. Lastly, once cured, the fish body is ready for operation **(3)**. This figure and caption are reproduced with permission from Marchese *et al.*^46^

The anatomically proportioned body of a fishlike robot developed by Marchese *et al.*^46^ and detailed in section 6.1 was also fabricated using a similar lamination-based casting process, and this process is detailed in Figure 12. Supply lines that connect the posterior actuator pair are embedded within the body during step 2 (Fig. 12–2).

### 4.2. Retractable pin casting

Retractable pin casting allows the relatively simple channel structure of the cylindrical body segment to be cast without lamination. This fabrication process is advantageous because it eliminates the rupture-prone seems between the channels and constraint layer seem in the ribbed morphology fabricated through lamination-based casting. Additionally, retractable pin casting is well-suited for the modular fabrication of multibody soft robots. Here, segments are individually cast and then concatenated together to form the robot. Specifically, in this section we demonstrate retractable pin casting in the context of fabricating a cylindrical manipulator.

**FIG. 13.**
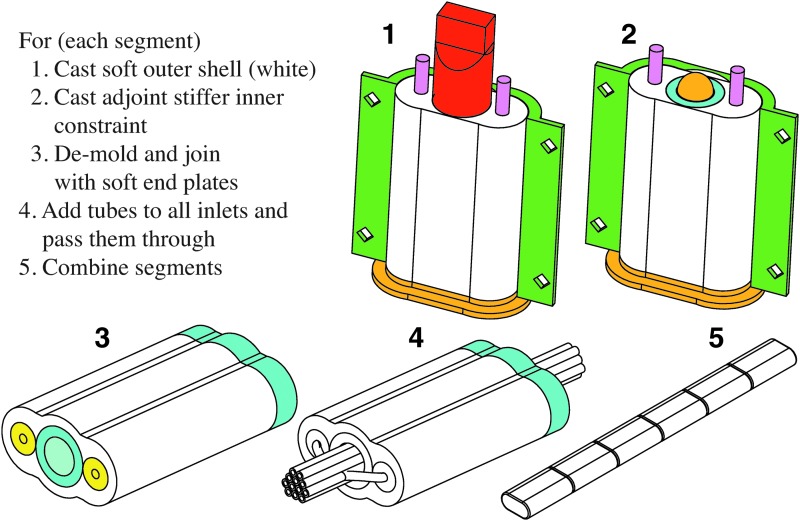
Fabrication process for the cylindrical manipulator morphology. Each body segment is casted using a two-step process where the outer soft layer **(1)** and inner stiffer layer **(2)** are poured. Once cured, the segments are joined to endplates using silicone adhesive **(3)**. Next, silicone tubing is connected to each embedded channel and the resulting tubing bundle is run inside each segment's hollow interior **(4)**. Lastly, the body segments are serially connected using adhesive to form the manipulator **(5)**. Color images available online at www.liebertpub.com/soro

A cylindrical manipulator, like that detailed in section 7.2, is fabricated through a retractable-pin casting using pourable silicone rubber^3,5^ and 3D-printed molds.^1^ Figure 13 details this process. First, each body segment is independently fabricated in steps 1–3, and later these segments are joined serially to form the arm in steps 4 and 5. To start, a four-piece mold is printed. The mold is then poured in two steps. In step 1, a low elastic modulus rubber is mixed,^3^ degassed in a vacuum,^4^ and poured to form the body segment's soft outer layer, shown in *white*. The mold's outer piece—one half of it is shown in *green*—functions to form the segment's exterior. Metal rods shown in *pink* are inserted into the mold and are held in place by the *orange* bottom piece of the mold. These rods will form the cavities for the segment's two lateral fluidic actuation channels. After the outer layer has cured, the *red* rigid sleeve is removed in step 2 from the extruded feature of the *orange* bottom piece of the mold. This produces a cavity into which a slightly stiffer rubber^5^ is poured, forming the segment's partially constraining inner layer, shown in *cyan*. The extruded feature of the *orange* bottom piece, shown by its *orange* end tip, functions to produce the segment's hollow interior core. In step 3, the body segments are removed from their molds and joined to rubber^5^ endplates, shown in *cyan*, using silicone adhesive.^6^ The small *yellow* channel inlets were added on one side of the *pink* metal pins during step 1. In step 4, soft silicone tubes^7^ are joined to each embedded channel's inlet. The resulting bundle of tubes is passed through each segment's hollow interior. Lastly, in step 5, multiple body segments are attached at their endplates using the same adhesive.^6^

### 4.3. Lost wax casting

As mentioned, existing soft robots are often produced through a multistep lamination process, which produces seams and is prone to delamination. By abandoning the need for lamination, the retractable pin fabrication process enables seamless channel structures; however, the channel structures are limited to a relatively simple shape. For these reasons, we introduce lost-wax casting as part of the fabrication process for soft actuators. With this, arbitrarily shaped internal channels can be achieved to enable a wider range of applications. As examples, in this section we fabricate a pleated unidirectional gripper and a ribbed soft fish tail using the lost-wax approach.

**FIG. 14.**
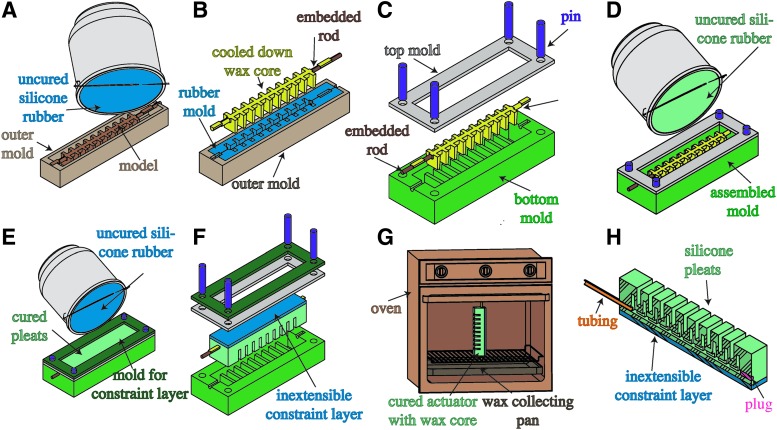
Fabrication process for the pleated actuator morphology: **(A)** pour and cure a rubber mold; **(B)** pour wax core with embedded supportive rod; **(C)** combine bottom mold, top mold, and wax core using pins; **(D)** pour rubber into assembled mold; **(E)** pour stiffer rubber on top of the cured actuator to form a constraint layer; **(F)** remove cured actuator from mold; **(G)** melt out wax core from the actuator using an oven; and **(H)** add silicone tubing and plug using silicone sealant.

The complete fabrication process for a pleated actuator consists of eight steps that are depicted in Figure 14. In step (A), harder silicone rubber^10^ is poured into a mold, which contains a 3D-printed model of the wax core. In preparation for step (B), the model is removed and the rubber mold is left inside the outer mold. Next, a rigid rod or tube, for example, made of carbon fiber,^12^ is used as a supportive inlay for the wax core. The rod is laid into the cavity of the rubber mold, supported on both ends by the outer mold. This ensures that the wax core does not break when removed from the rubber mold. Mold release spray is applied to the silicone rubber mold to ease the wax core removal process. The wax^11^ is heated up until it becomes fully liquefied. The assembly of the rubber mold and the outer mold is heated up for a few minutes to the same temperature as the wax. Using a syringe, the liquid wax is injected into the assembly. Within a few minutes, the injected wax will start to solidify and significantly shrink in volume; this is counteracted by injecting more hot wax into the solidifying wax core during the cool-down period. In step (B), the wax core is first allowed to completely cool down, then it is released from the mold. In step (C), the cooled down wax core is assembled together with the bottom mold, which defines the pleated structure of the actuator. The mold assembly is aligned with a top mold using pins. This top mold provides additional volume to cover the wax core. In step (D), low elastic modulus rubber^3^ is mixed, degassed in a vacuum,^4^ and poured to form the pleats and allowed to cure. In step (E), stiffer rubber is poured on top of the cured pleats to form a constraint layer. In step (F), the cured actuator is removed from the mold. In step (G), most of the wax core is melted out by placing the cured actuator into an oven in an upright position. After this, remaining wax residues are cooked out in a boiling water bath. Finally, in step (H) a silicone tube^9^ and a piece of silicone cord^13^ get covered with silicone adhesive^6^ and are inserted into the front and back holes, respectively. The actuator can be used as a unidirectional gripper (see Fig. 7) or as one agonist actuated segment within a multiple body manipulator (see section 7.3).

**FIG. 15.**
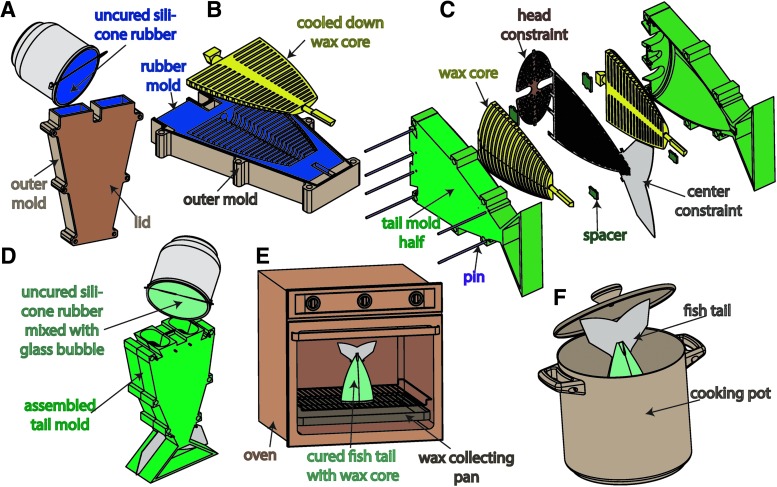
Fish tail fabrication process: **(A)** pour and cure a rubber mold; **(B)** pour wax cores; **(C)** combine head constraint, center constraint, and wax cores with tail mold halves; **(D)** pour rubber mixed with glass bubbles into assembled tail mold; **(E)** using an oven melt out wax core from the cured fish tail; and **(F)** cook out remaining wax to create desired actuator cavities.

The actuated body of the hydraulic fish detailed in section 6.2 is also produced via lost-wax casting. The fabrication process is depicted in Figure 15. In step (A), the rubber mold is poured and cured inside an assembly consisting of an outer mold with lid and a model for the core inside of it. In preparation for step (B), the lid and the model core are removed and the rubber mold is left inside the outer mold. The rubber mold receives a small carbon fiber tube as an inlay in its center cavity. This ensures that the wax core does not break when being removed from the rubber mold. Mold release spray is applied to the silicone rubber mold to ease the wax core removal process. The wax is heated up until it becomes fully liquefied. The assembly of rubber mold and outer mold is heated up for a few minutes to the same temperature as the wax. Using a syringe, the liquid wax is injected into the assembly. Within a few minutes, the injected wax will start to solidify and significantly shrink in volume; this is counteracted by injecting more hot wax into the solidifying wax core during the cool down. In step (B), the wax core is first allowed to completely cool down, then it is released from the mold. In step (C), a head constraint, a center constraint, and two wax cores are assembled together inside the tail mold halves using spacers, positioning pins and screws. In step (D), a mix of silicone rubber with glass bubbles is poured into the tail assembly and allowed to cure. In step (E), most of the wax core is melted out by placing the fish tail in an upright position into an oven. Finally, in step (F) the remaining wax residues are cooked out in a boiling water bath.

## 5. Power

Fluidic power sources present many challenges for soft robots. There are three major ways to characterize these power sources: by transmission fluid, circuit continuity, and portability.

### 5.1. Transmission fluids

Recently, Wehner *et al.* reviewed existing pneumatic energy sources.^73^ However, in general the actuators detailed in section 3.2 can be powered using either pneumatic or hydraulic systems where gases or liquids, respectively, are the transmission fluid. Pneumatics are advantageous for powering FEAs because they provide a low viscosity power transmission medium. High flows can be achieved with relatively low driving pressures. However, gases also introduce compressibility into the power transmission system, and these dynamics can be difficult to model (refer to Marchese *et al.*^49^) and can produce undesirable time delays. Hydraulics are advantageous because liquids are relatively incompressible when compared to gases, meaning power can be transferred almost immediately from the power source to the actuators. However, to achieve comparable volumetric flow rates, liquid drive systems often require high driving pressures and/or low impedance (large diameter) power transmission lines because of the increased viscosity of the transmission medium.

### 5.2. Circuit continuity

**FIG. 16.**
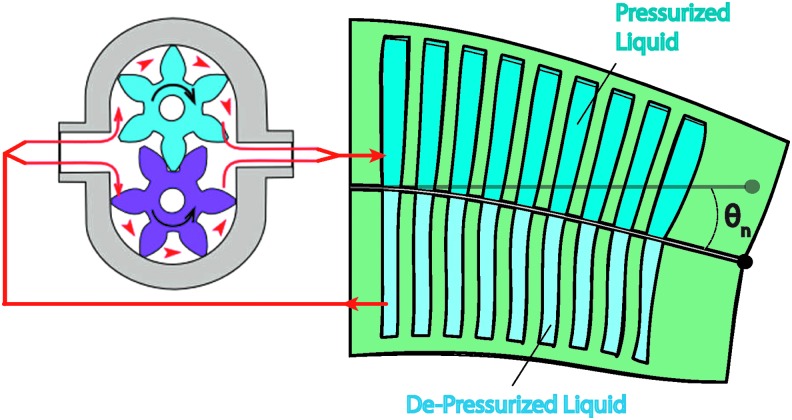
Closed-circuit power system used to drive actuation in the soft hydraulic fish. Color images available online at www.liebertpub.com/soro

Further, the actuators detailed in section 3.2 can be powered using either open-circuit or closed-circuit power systems. Open-circuit power systems exhaust the transmission fluid to the environment, whereas closed-circuit systems recover fluid delivered to the actuators. Open-circuit systems are advantageous because they do not require mechanisms to repressurize and return transmission fluid to the supply. However, they often rely on passively exhausting transmission fluid to ambient/environmental pressure, meaning the actuator depressurization is unactuated and is a function of the actuator's compliance and the impedance of the exhaust pathway. Please refer to Marchese *et al.* for examples of open-circuit power systems.^44,46^ Closed-circuit systems (see Fig. 16) are advantageous because the amount of transmission fluid is constant and moved around within the system; this means the power system's fluid medium is not required to match the operating environment (e.g., a soft robot fish powered by pneumatics swimming underwater). Furthermore, because the volume of transmission fluid is constant, the power system can typically vacuum fluid from the actuator under power; meaning the system has control authority over actuator depressurization. The disadvantage to closed-circuit systems is that they typically require additional plumbing to complete the fluid circuit and supporting hardware like a revisable pump. Please refer to references^8,9,48^ for examples of closed-circuit power systems.

### 5.3. Portability

The portability of a power source may be of significant interest to a soft roboticist. For example, locomotory soft robots are typically designed under the constraint of being self-contained, meaning all supporting hardware is located onboard the robot. Additionally, if the untethered robot is intended for high-speed maneuvers, then compressed gas^46^ or combustion^47^ are viable power alternatives. However, if prolonged operations are required, then open-circuit pumps^4,45^ or closed-circuit pumps^9^ are suitable options.

## 6. Locomotion

Soft and continuously deformable locomotion systems can be made from fluidic elastomer body segments. Specifically, in this section we detail how soft robotic fish can be composed by combining the actuated segments that were presented in section 3.2 with a portable power system.

### 6.1. Pneumatic fish

**FIG. 17.**
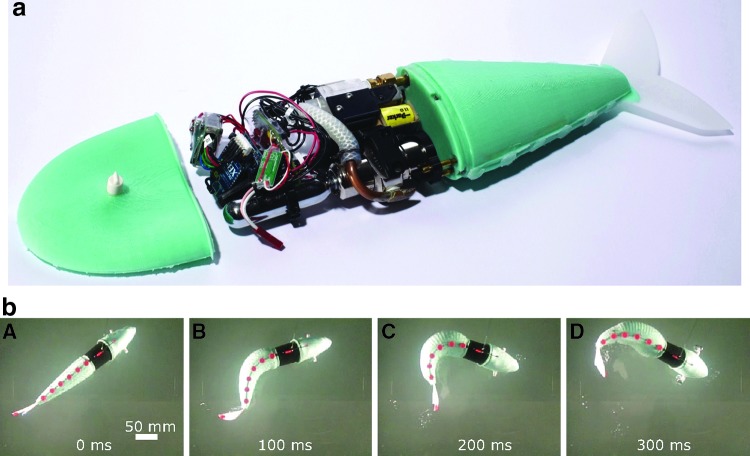
A soft pneumatic robotic fish. **(a)** An overview of the robotic system; photo courtesy of Devon Jarvis. **(b)** A sequence depicting the fish performing an escape response. Color images available online at www.liebertpub.com/soro

The soft pneumatic fish developed in Marchese *et al.*,^46^ with a ribbed actuator, is shown as a complete system in Figure 17a and performing an escape response in Figure 17b.

### 6.2. Hydraulic fish

**FIG. 18.**
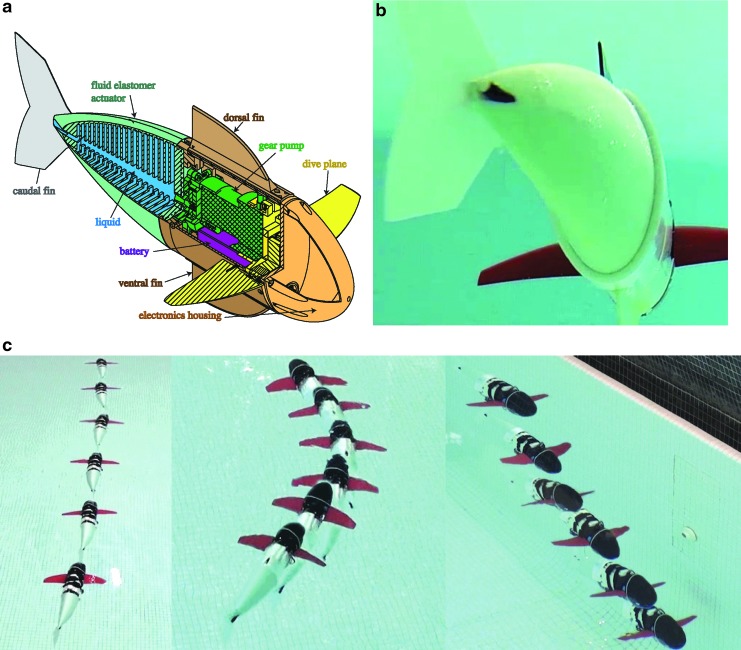
A soft hydraulic robotic fish: **(a)** a schematic of the system; **(b)** underwater swimming motion; and **(c)** example of continuous forward swimming, yaw motion, and diving.

The soft hydraulic fish^9^ with a single ribbed actuator is shown as a complete system in Figure 18a. A close-up view is shown in Figure 18b, and the 3D swimming capabilities are shown in Figure 18c.

## 7. Manipulators

Soft and continuously deformable manipulators can be assembled from bending fluidic elastomer segments. Specifically, in this section we detail how multisegment manipulators can be composed by serially concatenating the actuated segments that were presented in section 3.2.

### 7.1. Ribbed

**FIG. 19.**
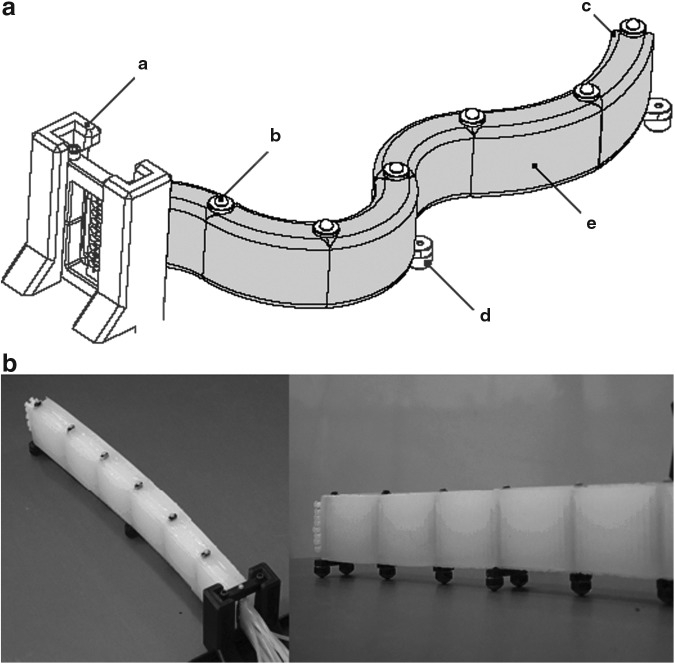
A ribbed soft manipulator prototype. **(a)** The arm is composed of homogeneous and independently actuated ribbed segments (e). The base of the arm's first segment is fixed (a) and the end of its last segment is the end-effector (c). Markers (b) identify the endpoints of each segment, and ball transfers (d) mitigate friction. **(b)** Photographs of the ribbed manipulator prototype.

Structurally, a ribbed arm is composed of serially concatenated, homogeneous ribbed body segments. By volume, over ninety-seven percent of the ribbed manipulator is soft silicone rubber, excluding the feet. This manipulator is depicted in Figure 19a and was initially developed in Marchese *et al.*^8^ The manipulator can theoretically be composed of any number of the aforementioned ribbed segments (Fig. 19a-e), but practically, we have constructed a six-segment prototype (Fig. 19b). All twelve fluidic transmission lines as well as channel-to-supply interfaces are embedded within the manipulator's center layer. Markers are located at the interface between segments (Fig. 19a-b), making segment endpoints identifiable to an external localization system. The starting point of the arm's first segment (Fig. 19a-a) is grounded to the platform on which the arm moves, and we refer to this as the base. Ball transfers (Fig. 19a-d) are also located at each segment endpoint to allow the arm to move on a two-dimensional plane with minimal friction. In many experiments conducted throughout this work, the pose of the arm's end-effector (Fig. 19a-c) is controlled.

### 7.2. Cylindrical

**FIG. 20.**
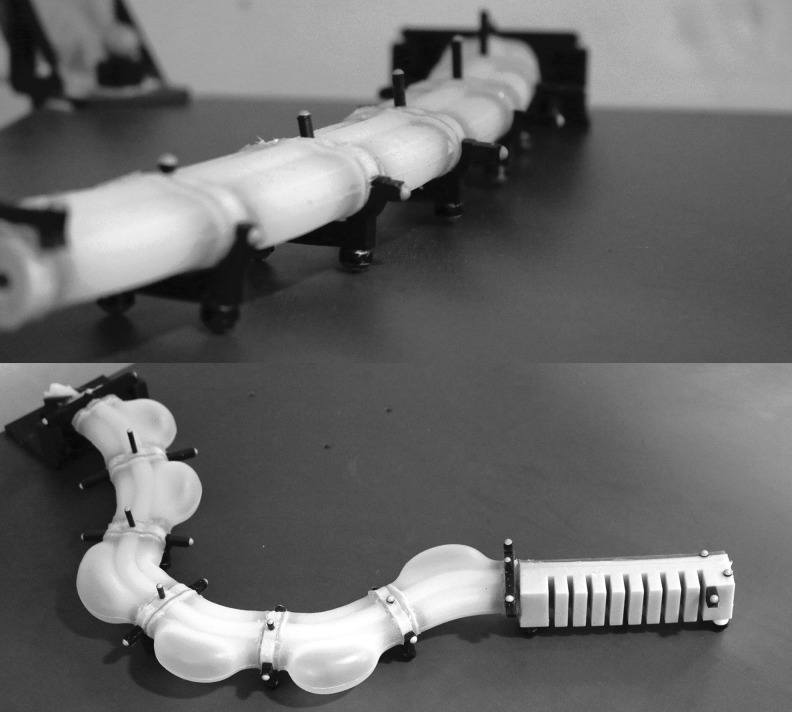
A planar cylindrical soft manipulator prototype with and without a pleated finger-like gripper.

We can also compose a manipulator from cylindrical fluidic elastomer segments, as shown in Figure 20, and initially developed in Marchese *et al.*^10^ Just as in the ribbed composition, cylindrical segments are joined end-to-end. Here, fluid transmission lines are passed through the manipulator's hollow center. This feature not only facilitates segment concatenation, but also allows for modular composition of a manipulator, because transmission lines are not permanently embedded within the elastomer. Additionally, this manipulator type is only composed of soft silicone rubber, as there is no inextensible constraint. No other materials are used, except for the attached ball transfers to mitigate ground friction.

**FIG. 21.**
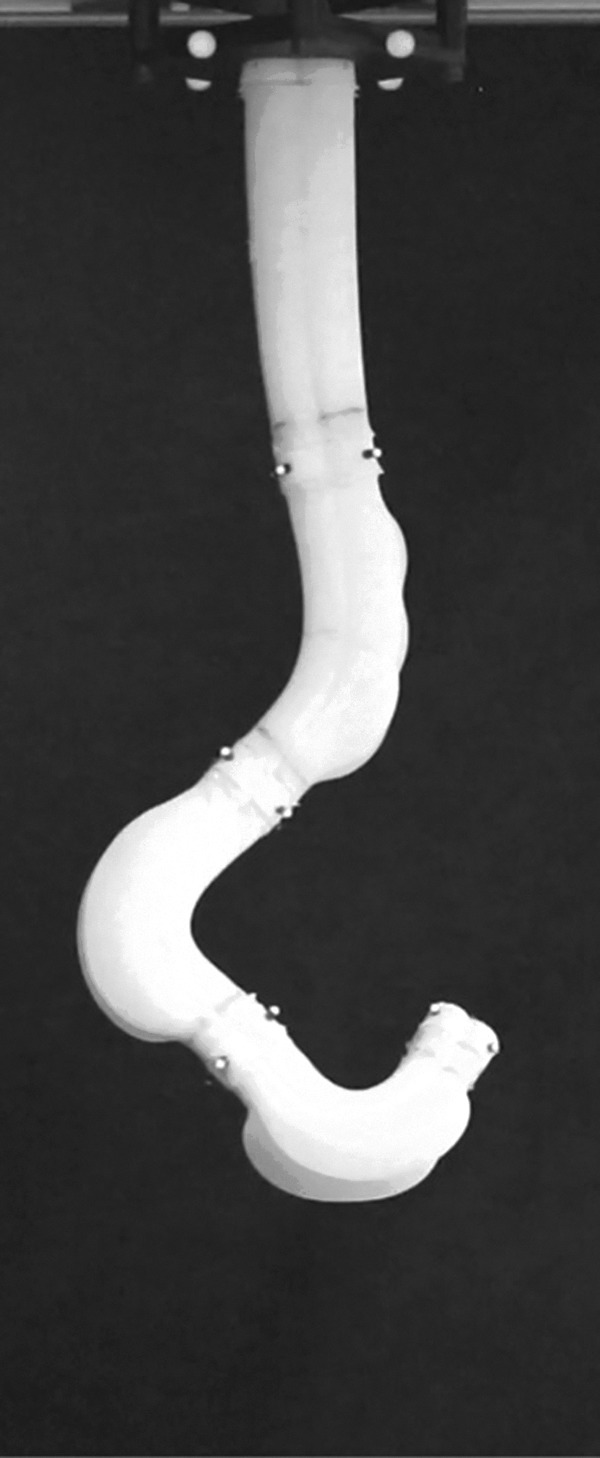
A spatial cylindrical soft manipulator prototype.

Additionally, using four actuated channels per body segment, we have created a multisegment spatial cylindrical manipulator in Marchese and Rus.^48^ This enables three-dimensional end-effector positioning and is shown in Figure 21.

### 7.3. Pleated

**FIG. 22.**
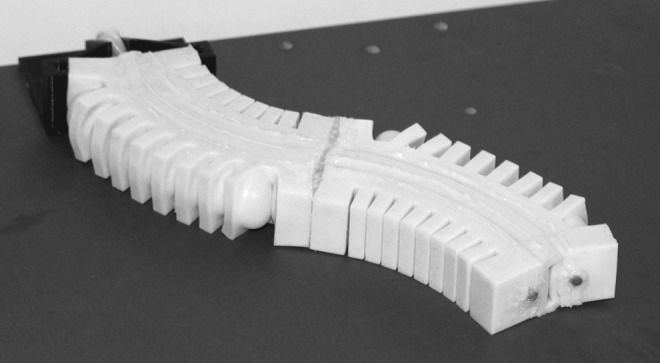
A pleated soft manipulator prototype, composed of two segments with two degrees of freedom each.

A manipulator can also be composed from pleated fluidic elastomer segments, as shown in Figure 22. Just as in the ribbed and cylindrical composition, pleated segments are joined end-to-end. The fluid transmission lines are passed through along the central axis of the segments. A supportive hollow profile can be added to combine two segments. This pleated design allows for modular composition of a manipulator, because transmission lines are not permanently embedded within the elastomer. Additionally, this type of manipulator is, like the cylindrical manipulator, composed entirely of soft silicone rubber.

## 8. Discussions

The actuator designs and their fabrication methods described in this article provide recipes for the rapid fabrication of modular soft robots with arbitrary body morphology.

We showed three fundamentally different fabrication processes and discussed their strengths and weaknesses when using them to build completely soft unit-modules that can be concatenated into multisegment manipulators or used for locomotion. The lamination and the lost-wax casting processes allow for the embedding of heterogeneous functional elements like constraint layers or tubes into a soft actuator. This facilitates the interfacing to pressure sources or other system components. The simplicity of the retractable pin fabrication method allows for rapid prototyping of simple fluidic elastomer actuators without the risk of failed lamination, and without the need for a wax core. The lost-wax casting allows for almost arbitrarily shaped pressurizable cavity structures, created as a monolithic body without weakening seams caused by a lamination technique.

Furthermore, an experimental characterization of each segment morphology was presented, analyzing and comparing the effects of fluid energy onto a segment's bend angle and tip force. It was seen that the pleated segment morphology is the stiffest, followed by the cylindrical, and then the ribbed. The cylindrical morphology has a prominent bend angle nonlinearity for low input volumes, but its behavior becomes almost linear for higher inflations. Based on this insight, easier control of this morphology can be achieved through prepressurization of a cylindrical segment. Furthermore, the cylindrical morphology requires the most amount of fluid energy to produce a given bend angle. The ribbed and pleated morphology behave very similar in bending. The pleated segment generally requires more fluid energy than both the ribbed and cylindrical morphologies to produce a tip force. However, the pleated segment can accommodate significantly higher input energies and therefore can reach the highest maximum tip force, useful when a more powerful manipulation is required.

This class of completely soft manipulator morphologies is very well-suited for tasks requiring: (i) interactions with humans and environments to be safe; (ii) uncertainty to be mitigated at the hardware level; (iii) continuous and dexterous deformation; and/or (iv) hardware to take an unstructured, amorphous form. For example, by making robots from soft elastic materials, with no sharp edges and relatively low link inertia, a robot's reliance on sensors and software for safety is reduced. The prospects for safe integrations between a robot and human are generally increased when the compliance of the material composing the machine match those of soft biological materials,^7^ and this feature is inherent to robots made of soft silicone elastomer.

## Notes

^*^ This subsection also appears in the author's related work.^48^
